# A Review of the Newly Recorded Genus *Proceroplatus* Edwards, 1925 (Diptera: Keroplatidae) in China with Two New Species, and Its Characterization and Phylogenetic Implication of Mitogenomes

**DOI:** 10.3390/insects16090883

**Published:** 2025-08-25

**Authors:** Qingyun Wang, Yi Zhu, Yefei Yu, Liwei Liu, Hong Wu, Junhao Huang

**Affiliations:** 1 National Joint Local Engineering Laboratory for High-Efficient Preparation of Biopesticide, Zhejiang A&F University, 666 Wusu Street, Lin’an, Hangzhou 311300, China; 2 Dapanshan National Nature Reserve Administration of Zhejiang Province, Panan 322300, China; 3 Zhejiang Museum of Natural History, No. 6 Westlake Cultural Square, Hangzhou 310014, China

**Keywords:** *Proceroplatus*, new record, new species, molecular identification, mitochondrial genome

## Abstract

*Proceroplatus* (Keroplatidae: Keroplatinae) is a distinct keroplatid group comprising 39 described species. These species are found worldwide, but none had previously been reported in China prior to this study. In this paper, *Proceroplatus* is recorded for the first time from China, along with two new species: *P. dapanshanus* **sp. n.** and *P. biemarginatus* **sp. n.**, which were collected from the southern region. Here, a worldwide distribution map of this genus is presented by species, including the new ones. Images and detailed morphological descriptions are provided for each new species, accompanied by molecular identification based on the standard mitochondrial cytochrome oxidase subunit I (COI) gene. To clarify the mitogenomic characteristics of *Proceroplatus*, the well-assembled and annotated mitogenome of *P. dapanshanus* was obtained and described in detail. The comparative analyses and phylogenetic tree indicate that the mitogenomic evolution of keroplatids is relatively conserved and influenced not only by mutation pressure but also by natural selection and other factors.

## Simple Summary

*Proceroplatus* is a keroplatid group usually having wings marked with diverse patterns. They exhibit diverse larval behaviors, with some being predaceous and linked to ant-plants, while others are phytophagous and damage orchid roots. This genus is distributed worldwide (except Antarctica); however, it remains unreported in China, and its mitogenome has not yet been studied. In this study, *Proceroplatus* is reported in China for the first time, with two new species detailed: *P. dapanshanus* **sp. n.** and *P. biemarginatus* **sp. n.** The comprehensive descriptions include images of diagnostic features of the adults, along with molecular identification. Furthermore, the well-assembled and annotated mitogenome of *P. dapanshanus* was obtained and described in detail. In comparison to other related groups, the codon usage of keroplatid mitogenomes is influenced not only by mutation pressure but also by natural selection and other factors. Meanwhile, the phylogenetic tree affirms the ancestral state of keroplatid mitogenomic gene arrangements. Overall, our findings confirm the existence of *Proceroplatus*, with two new species described in China for the first time, and clarify the genetic characteristics of its mitochondrial genome.

## 1. Introduction

The adults of *Proceroplatus* Edwards usually have wings adorned with stripes of various patterns, except for *P. kerteszi* Lane. So far, there has been a lack of comprehensive knowledge about their biological habits, with only a limited number of reports of their foraging behaviors. Some species of *Proceroplatus* are predaceous and associate with ant-plants during their larval stage [1,2,3]. Other species were deemed phytophagous, related to the root damage of the Dutch orchids [4]. Adults of an undescribed species of *Proceroplatus* were recently reported to be flower visitors [5].

The genus *Proceroplatus*, initially described in Ceroplatinae (=Keroplatinae), was later treated as a subgenus of *Platyura* and *Orfelia* before its generic status was restored [6,7,8,9,10,11]. It is now classified in the tribe Orfeliini under Keroplatinae [3,12].

Before this study, there were 39 recognized species, including two fossil species within *Proceroplatus*. They were recorded from all zoogeographical regions except Antarctica but were primarily distributed pantropically [3,13]. The most recently described species is a fossil from Dominican Miocene amber [13]. Most are found in the Neotropical Region (26), while others occur in the Afrotropical (8), Australasian (5), Oriental (4), Nearctic (3), and Palaearctic (1) regions.

In this study, *Proceroplatus* is recorded in China for the first time, with two new species described in detail: *P. dapanshanus* **sp. n.** and *P. biemarginatus* **sp. n.** Furthermore, their taxonomic status is firmly supported by molecular identification. The detailed descriptions include images of adults and anatomical features, as well as molecular identification. A worldwide distribution and checklist for this genus are also provided. Additionally, a high-quality mitogenome for *Proceroplatus* has been acquired. Based on the mitogenomic dataset, comparative analyses and phylogenetic reconstructions have been conducted between this genus and other closely related groups.

## 2. Materials and Methods

### 2.1. Morphological and Molecular Identification

All specimens in this paper were collected using a Malaise trap and then preserved in 85% ethanol. The individuals were observed under a SUNNY SZMN7045 stereo microscope (Ningbo Sunny Instruments Co., Ningbo, China). Following observation, they were dissected into segments and prepared on microscope slides in Euparal^®^ (Carl Roth GmbH + Co. KG, Karlsruhe, Germany). Images of the examined specimens were captured using a KEYENCE VHX digital microscope (Keyence Corporation, Osaka, Japan). The new species was delineated through comprehensive morphological comparisons with all known congeners within the genus. The world distribution map was made by ArcGIS 10.2. Morphological abbreviations followed those in Matile (1988, 1990) [14,15]. All specimens are now deposited at the Institute of Forest Protection, Zhejiang A&F University (ZAFU), Hangzhou, Zhejiang, China.

After observation, legs or thoraxes of the samples were utilized to extract total DNA using the TIANamp Genomic DNA Kit (Tiangen Biotech Co., Beijing, China). The mitochondrial cytochrome oxidase subunit I (COI) gene was then amplified and sequenced bidirectionally using standard primers [16,17]. All newly acquired COI sequences have been uploaded to the project “GNAT” in the BOLD system (http://v4.boldsystems.org, accessed on 1 September 2021) and GenBank (https://www.ncbi.nlm.nih.gov/, accessed on 30 October 2024). The outgroup taxa were chosen according to Mantič et al. (2020) [12], using publicly available COI sequences from GenBank (File S1). All COI sequences involved in this study were integrated and then aligned in Geneious Prime^®^ (version 21.0.7+6-LTS) using the MAFFT v7.490 plugin [18]. The maximum-likelihood (ML) tree was constructed in Geneious using the RaxML algorithm and the “GTR + GAMMA” model [19].

### 2.2. Mitochondrial Genome Assembly, Annotation, and Analysis

The Illumina^®^ HiSeq platform (Illumina Inc., San Diego, CA, USA) was used to construct genomic DNA libraries (350 bp insert size), with sequencing carried out on the Illumina HiSeq PE150 system. MitoZ 3.6.0 [20] was utilized to assemble and annotate the mitochondrial genome. First, the proper pairing of R1 and R2 reads was verified. The entire raw dataset was subsampled for mitogenome assembly by setting float 1 to 0, using the invertebrate mitochondrial genetic code. The Megahit assembler (MitoZ) was employed, with the k-mer value (not exceeding maximum read length) set to 59, 79, 99, 119, and 141. Mitogenome completeness was verified by the length and circularity of the assembled genome in the MitoZ assembly report. Lastly, non-target taxonomic sequences were filtered by specifying the taxonomic name as Arthropoda. Ultimately, a high-quality mitochondrial genome with annotations was obtained.

The secondary structures of tRNAs were predicted and constructed using the MITOS Web Server (http://mitos.bioinf.uni-leipzig.de/, accessed on 16 March 2025) [21] and visualized using VARNA v3-93 [22]. Protein-coding gene (PCG) sequences were extracted using PhyloSuite v1.2.3 [23,24]. The nucleotide composition of the complete mitochondrial genome and the PCGs was calculated using Geneious, employing the following skewness formulas: AT skew = [A − T]/[A + T], GC skew = [G − C]/[G + C] [25]. Relative synonymous codon usage (RSCU) for the PCGs was calculated and displayed by PhyloSuite v1.2.3, utilizing the invertebrate mitochondrial code table. Codon usage bias (CUB) indices were determined using CodonW 1.4.2 and PhyloSuite.

To understand the evolutionary dynamics of keroplatid mitogenomes, the ENc-plot, PR2-plot, and Neutrality-plot analyses were performed among these three keroplatid species. ENc (Effective Number of Codons) and GC3s (GC content at third codon sites) were used to evaluate CUB, and the ENc-plot analysis was conducted following the formula as follows: ENc = 2 + GC3s + 29/[GC3s^2^ + (1 − GC3s)^2^] [26]. The ENc values of all the PCGs (except for ATP8, <200 bp) were calculated for each keroplatid species [27]. A3, T3, C3, and G3 (Content of A, T, C, and G at the third codon location) were used to detect PR2 (Parity Rule 2) bias [28,29]. GC12 (average value of GC1 and GC2) and GC3, representing the GC content of codons 1, 2, and 3, were used to create a neutrality plot [30]. The statistical analysis was conducted using SPSS v. 26, employing the Wilson parameter and a two-tailed test. The plots were created using RStudio v1.6.0.

### 2.3. Phylogenetic Analyses

While this study focuses on species-level taxonomy within *Proceroplatus*, broader mitogenomic comparisons were conducted to: (1) clarify the taxonomic status and mitogenomic characteristics of *Proceroplatus*, and (2) characterize the mitogenomic synapomorphies of Keroplatidae (e.g., gene rearrangements, codon usage biases). Mitogenomes from 13 species were obtained across three closely related families: Mycetophilidae, Sciaridae, and Cecidomyiidae (File S2). All mitogenomic datasets were imported into PhyloSuite v1.2.3, where 13 PCGs and two rRNA genes were extracted. MAFFT v7.313 [18] was utilized to perform multiple sequence alignments, and Gblocks 0.91b was employed to eliminate unreliable comparison areas [31]. PartitionFinder2 [32] was used to evaluate the optimal partitioning schemes and substitution models, with branch lengths unlinked in the concatenated alignments. Model selection was set to the Bayesian information criterion (BIC), and the search method was set to “greedy”. All genes were divided into five partitions, with the best-fit model according to BIC being GTR+F+I+G4. The ML analysis was performed using IQ-TREE v. 1.6.8 [33], with ultrafast bootstrap approximation (UFBoot) and SH-aLRT branch test replicates set to 10,000. The Interactive Tree of Life (iTOL) v7 (https://itol.embl.de/, accessed on 4 June 2025) was utilized to enhance the results. The tree topology was optimized using the label and branch options in the iTOL platform. Colored ranges were then created to highlight distinct clades for each family. Finally, a gene order dataset was imported to annotate each species with its mitochondrial genome arrangement.

## 3. Results

### 3.1. Generic Characters

Based on the previous descriptions of this genus [3,4,6,13,34,35,36], the characteristics of this genus are as follows: The antennae vary from slightly to significantly flattened, or even pectinate. Scutum, scutellum, anepisternum, and laterotergite are setose. Wings pattern is usually present; vein Sc is shorter than half of R_1_; C exceeds the end of R_5_. Tibial bristles are arranged regularly. Male genitalia have gonostyli divided into 2–3 lobes or bearing lobes. Tergite 10 has a pair of cerci.

Among genera in Orfeliini, this genus closely resembles *Taulyrpa* Edwards in wing venation and male genitalia (gonostyli divided into two lobes), but it can be distinguished from the latter by the cerci. *Proceroplatus* has a pair of developed cerci, whereas *Taulyrpa* has only one cercus in tergite 10. Additionally, wing patterns are present in most *Proceroplatus* species, but they are absent in *Taulyrpa* [7,37,38,39]. In comparison to other Orfeliini genera, it can be easily identified by the following integrated morphological synapomorphies: (1) gonostyli divided into 2–3 lobes or bearing lobes, (2) two developed cerci, and (3) wing pattern (although it varies, most genera lack a wing pattern).

### 3.2. Species and Distribution

**Figure 1:**
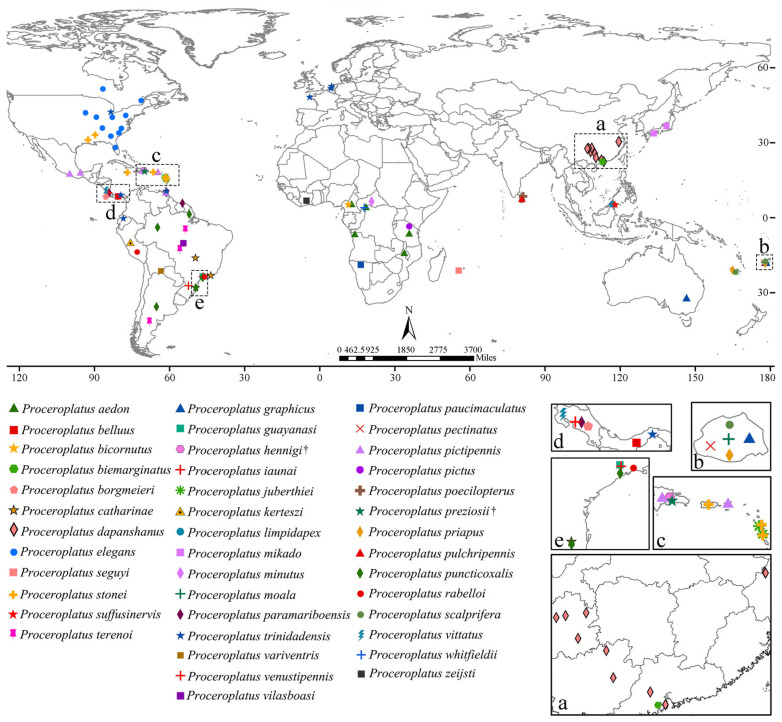
The worldwide distribution of *Proceroplatus* species. The five inserts magnify high-density regions where overlapping symbols obscure details (dashed boxes in the world map). † indicates that the species is a fossil species.

Currently, there are 42 recognized species within *Proceroplatus*, including two fossil species and two new species (File S3). They are widespread, and for the first time, two new species have been discovered in China. To clarify their distribution, we provide a world distribution map for this genus (Figure 1).

### 3.3. Key to the Species of Proceroplatus in Asia (Based on Adults)

1. Thorax with streaks on scutum ·····················································································································································································································2
- Thorax without streaks on scutum ·················································································································································································································4
2. Scutum with a pair of ill-defined linear streaks; terminalia black ··········································································································*P. pulchripennis* (Senior-White)
- Scutum with nonlinear streaks; terminalia brown ·······················································································································································································3
3. Two ocelli; scutum with a U-shaped dark streak; Sc reaching Costa; male cercus kidney-shaped ······································································*P. dapanshanus* **sp. n.**
- Three ocelli; scutum without U-shaped streak; Sc not reaching Costa; cercus suboval························································································ *P. biemarginatus* **sp. n.**
4. Head wholly black; a narrow stripe from middle of R_4+5_ to middle of M_1_····································································································· *P. suffusinervis* (Brunetti)
- Head brown or other colors; no stripe from middle of R_4+5_ to middle of M_1_············································································································································5
5. Costa reaching at or near 3/4 distance from R_4+5_ to M_1_··············································································································································································6
- Costa reaching at 1/2 distance from R_4+5_ to M_1_; wing tip with patterns·············································································································*P. poecilopterus* Edwards
6. Wings with three brown stripes················································································································································································*P. limidapex* (Edwards)
- Wings with a stripe and a rounded spot············································································································································································*P. mikado* (Okada)

### 3.4. Description of New Species

#### 3.4.1. *Proceroplatus dapanshanus* Wang *et* Huang **sp. n**.

**Figure 2:**
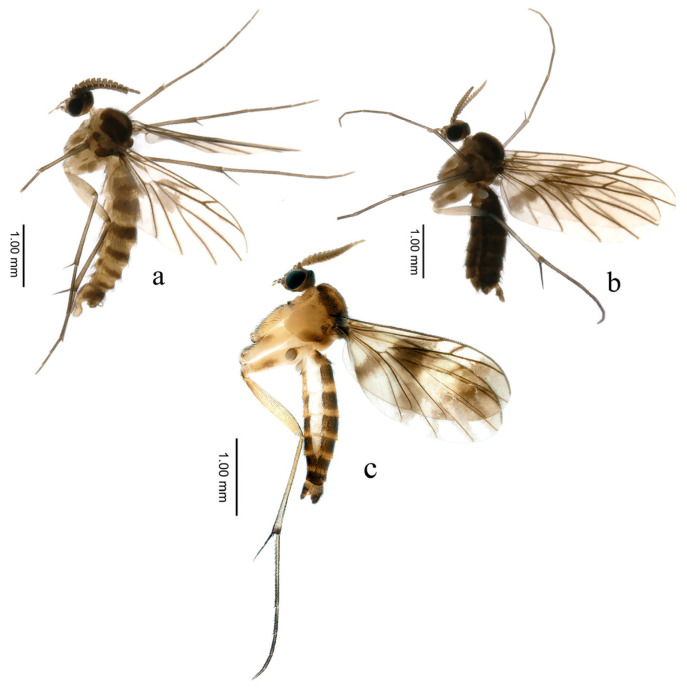
Adults of *Proceroplatus* spp. (**a**,**b**) *P. dapanshanus* **sp. n.**, lateral view. (**a**) holotype, male; (**b**) paratype, female. (**c**) *P. biemarginatus* **sp. n.**, holotype, male, lateral view.

**Figure 3:**
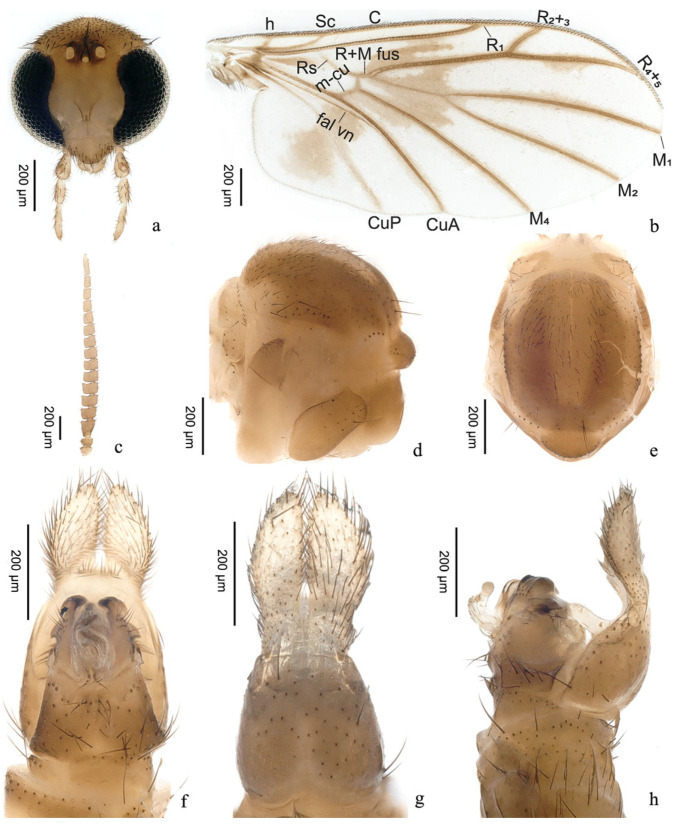
*Proceroplatus dapanshanus* **sp. n.**, paratype. (**a**) head (frontal view), antennae removed; (**b**) wing; (**c**) antenna; (**d**,**e**) thorax ((**d**) anterior view; (**e**) dorsal view); (**f**–**h**) terminalia ((**f**) ventral view; (**g**) dorsal view; (**h**) lateral view). Slide No. LWS-2-32, male.

urn:lsid:zoobank.org:act:05B6A1C5-2DD1-4DD9-ABAE-FC56EF8CA6D5
(Figure 2a,b and Figure 3)

**Diagnosis.** This new species resembles *P. mikado* (Okada, 1938) in wing vein patterns and genitalia, but can be distinguished by the absence of vein A_1_, the medial shade of the wing ending slightly before the wing margin, an oval spot at the intersection of R_2+3_ and R_4+5_, and a dark brown spot below the base of vein R_1_. In *P. mikado*, vein A_1_ exhibits a medial shade of the wing reaching the wing margin, along with a small spot near the intersection of R_2+3_ and R_4+5_, while the base of vein R_1_ has no spots. This new species can also be distinguished from congeneric species by its terminalia with a pair of kidney-shaped cerci.

**Type material. Holotype.** Male, CHINA: Zhejiang, Dapanshan National Nature Reserve (29.13° N, 120.61° E), 1,245 m, 16.VIII.2023, coll. Shuai Zhang, slide no. (DPS-2-1). Paratypes. 2 males and 10 females, the same data as the holotype, slides no. (DPS-2-2–DPS-2-13). Zhejiang: 31 males and 22 females, Longwangshan National Nature Reserve (30.23° N, 119.24° E), 671 m, 15.VII.2018, coll. Caixia Liu (LWS-3-1–LWS-3-5, LWS-2-30–LWS-2-32, LWS-2-43, LWS-2-51–LWS-2-53, LWS-2-55–LWS-2-95). 1 female, Tianmushan National Nature Reserve (30.19° N, 119.26° E), 361 m, 28.VI.2018, coll. Caixia Liu, slide no. TMS-2-50. Guangdong: 4 males and 4 females, Mt. Dinghu (23.15° N, 112.54° E), 56 m, 12.VI.2020, coll. Lei Qi, slide no. (DHS-8-62, DHS-8-74, DHS-8-75, DHS-8-79, DHS-8-86–DHS-8-89). 7 males and 3 females, Mt. Wugui (22.43° N, 113.44° E), 114 m, 16.VIII.2020, coll. Lei Qi, slide no. (WGS-9-5, WGS-9-6, WGS-9-9, WGS-9-16, WGS-9-18–WGS-9-23). Guangxi: 2 males, Dayaoshan National Nature Reserve (24.14° N, 110.19° E), 1520 m, 08.VI.2020, coll. Tao Li, slide no. (DYS-10-64, DYS-10-67). 1 female, Huaping National Nature Reserve (30.44° N, 110.00° E), 750 m, 15.VII.2020, coll. Lei Qi, slide no. (GXHP-40). Guizhou: 2 females, Mt. Leigong (26.36° N, 108.16° E), 680 m, 17.VII.2019, coll. Changlie Yang, slide no. (LGS-10-40, LGS-10-41). 2 males, Mt. Fanjing (27.91° N, 108.70° E), 750 m, 04.VII.2018, coll. Fanliang Liu, slide no. (FJS-10-29, FJS-10-31). 1 female, Meitan County (27.74° N, 107.46° E), 350 m, 15.VI.2019, coll. Bing Zhang, slide no. (GLG-8-4). 2 males, Mt. Honghuagang (27.64° N, 106.89° E), 550 m, 13.VI.2014, coll. Liang Ma, slide no. (HHG-7-90, HHG-7-91).

**DNA barcode.** The DNA barcode sequences from the holotype and paratype are deposited in BOLD. The BIN number is AEJ2594 in BOLD.

**Description:**

**Male** (Figure 2a). Body length (without antennae) 2.84 mm. Wing length 2.40 mm, width 0.94 mm.

Head (Figure 3a) brown, wider than long. Vertex with numerous dark setae on surface. Frons and face bare. Three ocelli in a triangular position, placed on slightly raised dark brown ocellar tubercle, laterals larger than median ocellus. Compound eye with slight invagination medially, covered with abundant pubescence. Clypeus inverted triangular, rounded apically, bearing several dark bristles posteriorly and laterally. Labellum beyond level of apex of clypeus, with bushy cilia. Maxillary palpus with four segments: first segment almost 3 times as long as wide; second segment with a sensory pit ventrally; third segment as long as second; fourth segment long and slender, almost 1.5 times as long as penultimate segment. Antenna (Figure 3c) brown and compressed; scape and pedicel with several subapical and apical setae; flagellum with 14 flagellomeres, ventral macrotrichia longer than dorsal setae; first segment nearly 1.5 times as long as second; segments 2–10 wider than long; segments 11–13 slightly longer than wide; terminal segment gradually narrowing, longer than first segment.

Thorax (Figure 3d–e) brown. Scutum brown, with a U-shaped dark streak, covered with numerous setae. Scutellum with row of setae at apex. Prothoracic spiracle bare. Anepisternum brown, with group of setae on hind part. Katepisternum pale brown, bare. Laterotergite brown, bearing several dark bristles on anterior half. Mediotergite dark brown, bare. Halter length 0.31 mm, stem hyaline, knob brown, setose.

Wing (Figure 3b) brown, with dark brown shade from cell r1 suffused through M_1+2_ stem, continuing to diffuse, reaching CuP, ending far from wing margin. Wing membrane covered with numerous microtrichia. Vein base of R_1_ with dark brown spot below. All veins thick, reaching wing edge. Vein Sc reaching C slightly beyond origin of Rs. Vein CuP, Rs, and distal half of m-cu paler, bare. Vein R_2+3_ and Sc bare. Vein A_1_ absent. Costa slightly more than half as long as length between R_4+5_ and M_1_. Vein R+M fusion about 1/7 of vein M_1+2_ stem in length. Vein m-cu longer than vein R+M fusion. Vein M_1_ about 3 times as long as vein M_1+2_ stem.

Legs (Figure 2a) brown, densely covered with dark setae. Tibiae with only inner spur; mid and hind tibiae spurs longer than fore tibiae. Fore tibiae with brown anterior comb; mid tibiae with black anterior comb; hind tibiae with black anterior and posterior combs. Fore tarsi almost twice length of tibiae; mid and hind tarsi almost 1.5 times length of tibiae.

Abdomen (Figure 2a) pale brown to brown, with bushy dark setae. Tergite 1 brown; tergite 2 with basal 3/4 brown; distal 1/4 pale brown; tergite 3 with basal 1/4 pale brown; distal 3/4 brown; tergites 4–8 brown.

Terminalia (Figure 3f–h) brown. Sternite 9 subtrapezoidal, with an emargination on posterior margin. Tergite 9 broad, subtrapezoidal, bearing abundant bristles. Tergite 10 with pair of kidney-shaped cerci, expanding medially, longer than tergite 9, covered with numerous setae on surface. Aedeagus hyaline, curved medially, rounded apically, dark brown, having several setae outside.

**Female** (Figure 2b). Antennae somewhat slender, except for genitalia; abdomen darker than in male individuals.

**Etymology.** The name of this new species derives from its holotype locality (Dapanshan National Nature Reserve), aiming to commemorate the great progress of biodiversity conservation efforts over the past two decades since the establishment of the Dapanshan National Nature Reserve Administration.

#### 3.4.2. *Proceroplatus biemarginatus* Wang *et* Huang **sp. n**.

**Figure 4:**
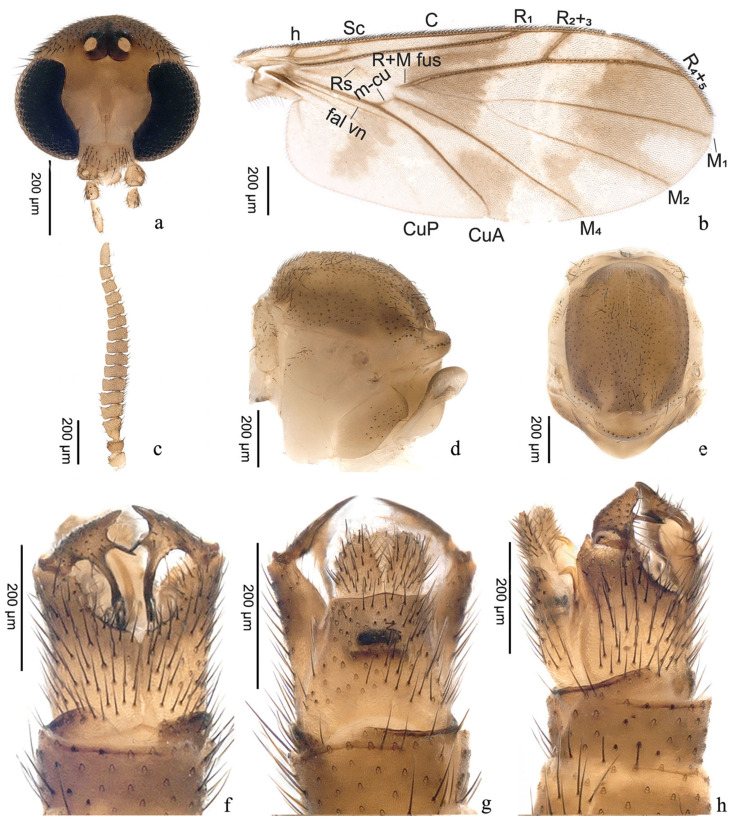
*Proceroplatus biemarginatus* **sp. n.**, holotype. (**a**) head (frontal view), antennae removed; (**b**) wing; (**c**) antenna; (**d**,**e**) thorax ((**d**) anterior view; (**e**) dorsal view); (**f**–**h**) terminalia ((**f**) ventral view; (**g**) dorsal view; (**h**) lateral view). Slide No. NL-9-48.

urn:lsid:zoobank.org:act:C384DFED-2443-4666-BCFF-A3FA6F7E752E
(Figure 2c and Figure 4)

**Diagnosis.** This new species is similar to *P. juberthiei* Matile, 1982 in wing and gonostylus patterns, but differs as follows: absence of spots at the start of Rs; terminalia with two horizontal streaks dorsomedially; cercus rounded apically; middle lobe widely separated from the outer lobe. In *P. juberthiei*, the start of Rs features a rectangular spot extending to CuA. The terminalia lacks any streaks, the cercus is trapezoidal apically, and the middle lobe is close to the outer lobe.

**Type material. Holotype. Male.** CHINA: Guangdong, Mt. Nanling (24.93° N, 113.00° E), 1210 m, 06.VII.2020, coll. Lei Qi, slide no. NL-9-48. **Paratype**. 1 male, the same data as holotype (NL-9-52).

**DNA barcode.** The DNA barcode sequences from the holotype and paratype are deposited in BOLD. The BIN number is AEJ8294 in BOLD.

**Description. Male**. Body (Figure 2c) length (without antennae) 3.42 mm. Wing length 1.97 mm, width 0.80 mm.

Head (Figure 4a) subcircular, brown. Vertex with dense dark setae. Frons and face bare. Two ocelli, placed on raised dark brown ocellar tubercle. Compound eye with emargination medially, covered with dense setae. Clypeus inverted triangular, bearing countable dark setae on surface. Labellum extending beyond apex of clypeus, with dense pubescence. Maxillary palpus with four segments: first segment shorter, with few dark brown setae; second segment subspherical, with ventral sensory pit; third segment nearly 1.2 times as long as wide; last segment suboval, almost 1.6 times as long as penultimate segment. Antenna (Figure 4c) brown and compressed; scape and pedicel with several subapical and apical setae; flagellum with 14 flagellomeres, lateral microtrichia sparse, dorsal macrotrichia longer than ventral setae; first segment elongated, about twice as long as second; segments 2–12 much wider than long; segment 13 slightly longer than wide; terminal segment as long as first.

Scutum (Figure 4d–e) brown, with two light brown streaks medially, dark brown laterally and apically, covered with bushy dark setae. Scutellum with several setae at apex. Prothoracic spiracle bare. Anepisternum pale brown, with a group of dark macrotrichia. Katepisternum pale brown, bare. Laterotergite brown, bearing countable setae on anterior part. Mediotergite brown, bare. Halter length 0.41 mm, stem translucent, knob brown, bearing setae.

Wing (Figure 4b) brown, with dark brown pattern of infuscations. First band of infuscation from apex of R_1_ to base of M_4_, uninterrupted, widening at contact with veins; cell cup with slightly brown shade medially, ending far from wing margin; second band of infuscation from apex of R_4+5_ to posterior half of CuP, uninterrupted, with apical U-shaped clear area in cells m1, m2 and cua. Microtrichia on numerous wing membrane. Costa almost reaching 0.7 distance from vein R_4+5_ to vein M_1_. Vein Sc incomplete, not reaching C. Vein Rs, m-cu, CuP, M_1+2_ stem, A_1_ and base of M_1_, M_2_ paler. Vein R+M fusion almost 1/5 of vein M_1+2_ stem in length. Vein M_1_ almost 3.2 times as long as vein M_1+2_ stem.

Legs (Figure 2c) brown. Hind coxae with several setae on posterior part, outer spur of hind tibiae almost six times as long as inner, hind tarsi near 1.4 times length of tibiae. Fore and mid legs damaged, only with coxae.

Abdomen (Figure 2c) pale brown to brown, covered with thick dark setae. Tergite 1 brown, tergites 2–5 with anterior 2/3 brown, posterior 1/3 pale brown, tergites 6–7 brown.

Terminalia (Figure 4f–h) brown. Tergite 9 longer than wide, anterior margin with U-shaped emargination; with two horizontal streaks dorsomedially, first stripe black, second stripe longer than first, dark brown. Cercus suboval, rounded apically. Gonocoxites much longer than gonostylus, with wide U-shaped ventral notch; anterior half brown, posterior half dark brown. Gonostylus with three apical lobes: outer lobes longest, about 2.5 times as long as medial, basal half brown, distal half dark brown, rounded bare; median lobe slender, dark brown, slightly longer than inner, pointed apically; inner lobe sub coniform, brown, pointed at apex.

**Etymology.** The name of this new species originates from the Latin words *bi* (two) and *emarginatus* (emarginate), designating the U-shaped emargination on the gonostylus.

### 3.5. Molecular Identification

**Figure 5:**
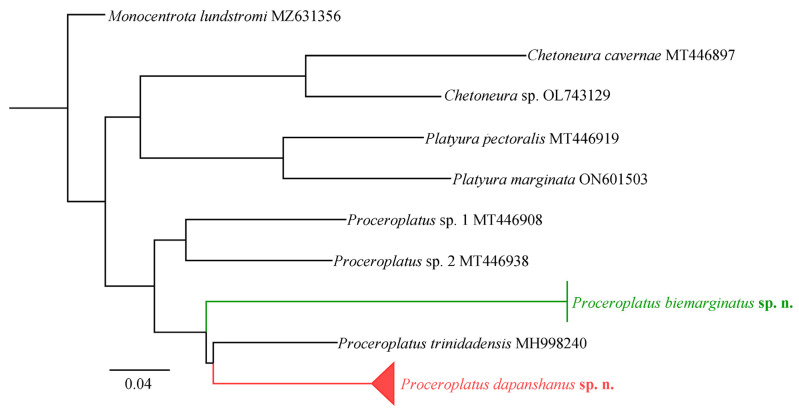
A maximum likelihood (ML) tree of *Proceroplatus*, terminal branch ends with the species name and GenBank accession number. The colored threads indicate these two new species.

The ML tree (Figure 5) revealed that each sample branch belonged to the monophyletic group of *Proceroplatus*. Furthermore, the new species, *P. biemarginatus* **sp. n.** (NL-9-48, NL-9-52), shows no genetic distance among its respective samples. The new species, *P. dapanshanus* **sp. n.**, exhibited a genetic distance ranging from 0 to 0.0153 among its samples. Additionally, all species within *Proceroplatus* displayed a genetic distance of 0 to 0.1669 from one another. In summary, these molecular identifications provide strong support for classifying the two new species within this genus.

### 3.6. Mitogenomic Characteristics

#### 3.6.1. Base Composition

The newly assembled mitogenome of *P. dapanshanus* is 15,688 bp in length, with a GC content of 21.003%, indicating a GC skew of −0.185. Among these three keroplatid mitogenomes, this species has a GC content similar to *Orfelia* sp. but significantly higher than that of *A. flava* in both mitogenomes and PCGs. The GC skews of the mitogenomes are relatively close. However, the AT skew of this species (0.005) is nearly zero. For PCGs, *P. dapanshanus* has a total length of 11,193 bp and a GC content of 23.184%, with a GC skew of −0.002 and an AT skew of −0.162, which are comparable to those of the other two keroplatids (Table 1).

#### 3.6.2. tRNA Structure Prediction

The mitogenome of *P. dapanshanus* consists of 22 tRNAs, with lengths ranging from 64 bp to 72 bp. The longest is trnV (72 bp), while the shortest is trnH (64 bp). The cumulative length of all tRNAs is 1477 bp, with an overall AT content of 82.6%. The prediction of secondary structures shows that most bases conform to canonical pairings, although a subset exhibits G-U mismatches. Except for Ser1, which lacks the DHU arm, all remaining tRNAs have a typical cloverleaf structure (File S4).

#### 3.6.3. Relative Synonymous Codon Usage (RSCU)

**Figure 6:**
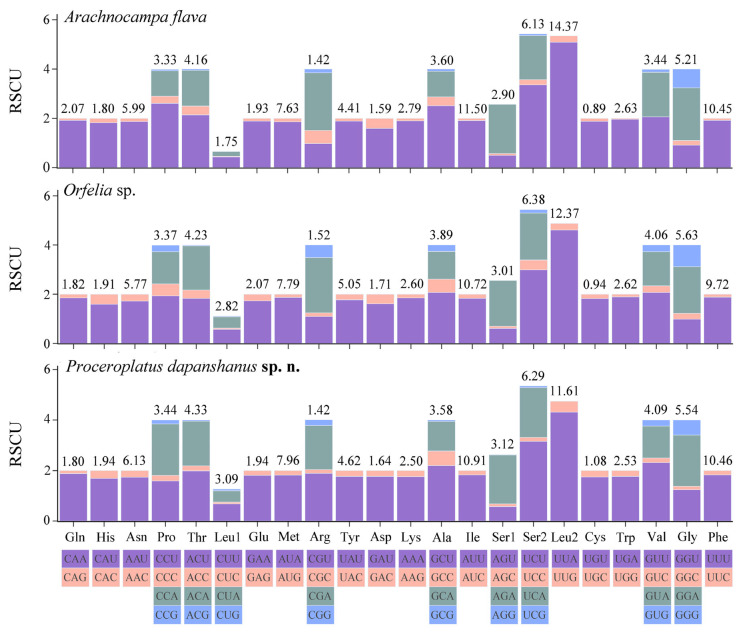
Relative synonymous codon usage (RSCU) among three keroplatid species. Values at the top of the bars indicate amino acid usage.

For the mitogenome of *P. dapanshanus*, CUB is evident in all synonymous codons, with optimal codons tending to end with A or U (T). Compared to the other two keroplatid species, the mitogenomic CUB of this new species is notably more pronounced. There are 28 high-frequency codons (RSCU > 1), accounting for 45.16% of all codons. By contrast, there are 26 and 27 high-frequency codons in the mitogenomes of *A. flava* and *Orfelia* sp., respectively. Nevertheless, the codon usage patterns are highly similar among the mitogenomes of these keroplatids. They all share 62 common codons (excluding two stop codons), among which UUA, AUU, and UUU are used most frequently in succession. Furthermore, their mitogenomes exhibit a significant A/U bias at the third nucleotide position of codons as well (Figure 6).

#### 3.6.4. ENc-Plot, PR2-Plot and Neutrality-Plot Analyses

**Figure 7:**
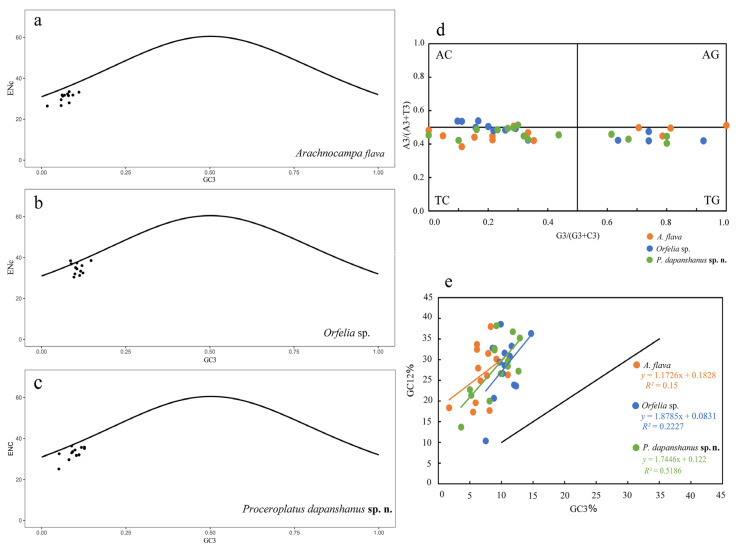
ENc-plot, PR2-plot, and Neutrality-plot analyses of the mitogenomic protein-coding genes (PCGs) for *Proceroplatus dapanshanus* **sp. n.**, *Orfelia* sp., and *Arachnocampa flava*. (**a**–**c**) Enc plots (except ATP8), with theoretical curves. (**d**) PR2 plot, with center point (X = 0.5, Y = 0.5). (**e**) Neutrality plot, with regression curve equations and *R*^2^ values.

For the mitogenome of *P. dapanshanus*, the ENc values range from 25.21 (ND4L) to 36.30 (ATP6), with COIII (35.65), CYTB (35.76), and ND5 (35.12) being the only ones slightly above 35. All observation points fall below the theoretical curve, indicating a significant deviation. For the other keroplatid mitogenomes, the ENc values (26.67–33.43) for *A. flava* are all below 35, with all observation points significantly falling beneath the theoretical curve. In contrast, the values for *Orfelia* sp. are comparatively higher (30.44–38.55), with most observation points remaining below the theoretical curve (Table 2, Figure 7a–c).

The PR2 plot shows that most of the PCG points cluster in the third quadrant, followed by the fourth quadrant, although they are positioned far from the central point for *P. dapanshanus*. They closely resemble the PCG locations of *A. flava*, but they differ somewhat from those of *Orfelia* sp., as three PCGs fall completely into the second quadrant for the latter. Nevertheless, all observation points are located around the central horizontal axis (Y = 0.5) but extend away from the center point (Figure 7d, File S5).

The neutrality plot indicates that all PCG points lie above the diagonal of the coordinate axes (GC3 = GC12), with the slope of the regression curve being 1.745 and an *R^2^* value of 0.519 for *P. dapanshanus*. For the other two keroplatids, their PCGs are also situated above the diagonal, with the regression curves exhibiting slope values of 1.173 and 1.879, along with *R^2^* values of 0.150 and 0.223 for *A. flava* and *Orfelia* sp., respectively (Figure 7e, File S5).

### 3.7. Phylogeny and Gene Arrangements

**Figure 8:**
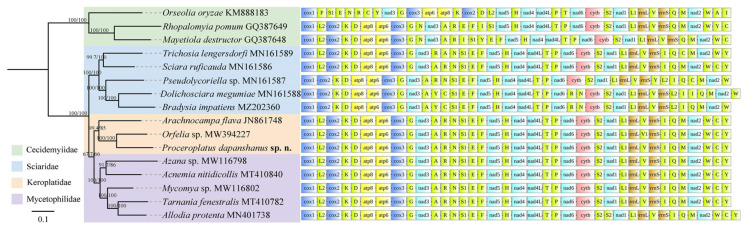
The maximum likelihood (ML) tree of 16 species from four families within Sciaroidea, reconstructed using the concatenated sequence matrix of 13 protein-coding genes (PCGs) and two ribosomal RNAs (rRNAs) genes. Support values (SH-aLRT/UFBoot) are listed on each node. Mitogenomic gene arrangements are annotated to each terminal branch.

In the ML tree, the branch of *P. dapanshanus* converges with other keroplatids, forming a monophyletic keroplatid clade that clusters with the separate mycetophilid clade, followed successively by the independent sciarid and cecidomyiid clades. All the aforementioned clades are highly supported (SH-aLRT > 99, UFBoot ≥ 85) in the topological tree. The arrangements of mitogenomic genes are consistent within each taxon of the first two clades. In contrast, the orders of mitogenomic genes exhibit significant variation across nearly all taxa in the latter two clades, except for the mitogenome of *Sciara ruficauda*, whose gene arrangements remain consistent with those of keroplatids and mycetophilids (Figure 8).

## 4. Discussion

The taxonomic statuses of these new species were confirmed through their morphological characteristics and molecular identification, as well as the classification status of the newly recorded genus *Proceroplatus* based on mitogenomic phylogenetics. This also aligns with the previous molecular phylogenetic study that utilized only gene markers [12].

Lower ENc values (<35) indicate higher codon bias [26]; a similar pattern exists for keroplatid mitogenomes, suggesting a strong CUB. The ENc standard curve represents the expected relationship between ENc (Effective Number of Codons) and GC3s (GC content at third codon positions) under the null hypothesis that codon usage bias (CUB) is determined solely by mutational pressure, without influence from natural selection or other factors. Most PCGs are located below the ENc standard curve and away from it, indicating that their CUB is influenced not only by mutation pressure but also by natural selection and other factors to a great extent. Furthermore, natural selection may play a major role in influencing their codon bias [40,41]. The distribution of PCGs across the PR2 plot reveals a significant bias in AT usage at the third codon position [28,29]. This may arise from the asymmetric interaction between mutation pressure and natural selection [41]. The neutrality plot analysis indicates that GC content is significantly lower at the third codon position compared to the first and second positions, implying a strong bias in AT usage. The combined evidence from ENc analysis, PR2 bias, and neutrality plots demonstrates that codon usage in keroplatid mitogenomes is shaped by a complex interplay of mutation pressure (driving AT bias), natural selection (maintaining functional and translational optimization), and other factors. Consequently, the codon usage of keroplatid mitogenomes is influenced not only by mutation pressure but also by natural selection, as well as other factors [41,42,43].

The previous mycetophilid mitogenomic research demonstrated that gene orders are conserved in Mycetophilidae and Keroplatidae; however, only one keroplatid mitogenome (*A. flava*, belonging to Arachnocampinae) was included [44]. The conserved gene order “*cox1*-*L2*-*cox2*-*K*-*D*-*atp8*…” found in keroplatids aligns with the ancestral insect mitogenome, confirming the ancestral state of keroplatid mitogenomic gene arrangements by incorporating two additional representative species, *P. dapanshanus* and *Orfelia* sp., classified within Keroplatinae [45,46].

## 5. Conclusions

This study reports the first occurrence of *Proceroplatus* in China, describing two new species (*P. dapanshanus* and *P. biemarginatus*) with detailed morphological diagnoses and molecular characterization. The mitogenome of *P. dapanshanus* reveals conserved gene arrangement patterns in Keroplatidae, supporting their ancestral status among related dipteran groups. Codon usage analyses reveal that natural selection, alongside mutation pressure, shapes mitogenomic evolution in this family. The discovery expands the known distribution of *Proceroplatus* and provides foundational genetic data for future systematic studies. Our integrative approach, combining morphology, DNA barcoding, and mitogenomics, establishes a robust framework for species delimitation and phylogenetic placement within this ecologically diverse genus.

## Figures and Tables

**Table 1:** Mitogenomic characteristics of three keroplatid species.

Species	A+T%	G+C%	AT-Skew	GC-Skew	Length (bp)
	Mitogenome				
*Arachnocampa flava*	82.013 78.912 78.997	17.987	−0.034	−0.183	16,923
*Orfelia* sp.		21.088	0.025	−0.188	15,521
*Proceroplatus dapanshanus*		21.003	0.005	−0.185	15,688
	**Protein coding gene (PCG)**				
*Arachnocampa flava*	78.932 76.277 76.816	21.068	−0.164	0.006	11,202
*Orfelia* sp.		23.723	−0.152	−0.003	10,863
*Proceroplatus dapanshanus*		23.184	−0.162	−0.002	11,193

**Table 2:** The GC3s and ENc values of 12 protein-coding genes (PCGs) from the mitogenomes of three keroplatid species.

	Species	*Arachnocampa flava*		*Orfelia* sp.		*Proceroplatus dapanshanus*	
PCG		GC3	ENc	GC3	ENc	GC3	ENc
**ATP6**		0.092	31.80	0.086	38.43	0.089	36.30
**COI**		0.083	33.43	0.099	32.02	0.092	33.62
**COII**		0.079	31.49	0.105	37.38	0.089	32.99
**COIII**		0.061	31.80	0.147	38.55	0.118	35.65
**CYTB**		0.061	29.50	0.116	33.38	0.129	35.76
**ND1**		0.063	31.25	0.113	31.26	0.111	32.09
**ND2**		0.059	26.67	0.123	32.49	0.052	32.53
**ND3**		0.110	33.17	0.097	30.44	0.110	31.77
**ND4**		0.077	32.32	0.101	35.09	0.099	34.37
**ND4L**		0.081	27.98	0.120	36.08	0.050	25.21
**ND5**		0.067	31.70	0.105	34.47	0.127	35.12
**ND6**		0.017	26.44	0.088	36.90	0.081	29.73

## Data Availability

The original contributions presented in this study are included in the article. Further inquiries can be directed to the corresponding authors.

## Supplementary Materials

The following supporting information can be downloaded at: https://www.mdpi.com/article/10.3390/insects16090883/s1. File S1: BIN ID and GenBank accession numbers of all cytochrome oxidase subunit I (COI) gene sequences used in this study; File S2: GenBank accession numbers of sciaroid mitogenomes used in this study; File S3: World checklist of *Proceroplatus*; File S4: Prediction of tRNA secondary structures in the mitogenome of *Proceroplatus dapanshanus* **sp. n.**; File S5: ENc-plot, PR2-plot, and neutrality-plot results of three keroplatid mitogenomes.
