# Supplementary material for: A Review of the Newly Recorded Genus Proceroplatus Edwards, 1925 (Diptera: Keroplatidae) in China with Two New Species, and Its Characterization and Phylogenetic Implication of Mitogenomes
Source: Insects. 2025 Aug 25;16(9):883. doi: 10.3390/insects16090883 (PMC12471120; doi:10.3390/insects16090883)
Supplement: Supplementary file 1 [file insects-16-00883-s001.zip › File S1.pdf]

File S1. BIN ID and GenBank accession numbers of all cytochrome oxidase subunit I (COI) gene sequences used in this study.

Species	Sample ID	BIN ID	GenBank No.	Reference
Chetoneura sp.	JSK59b	AEG7736	OL743129	Ševčík *et al.*, 2021
Chetoneura cavernae	JSK9	AEG7737	MT446897	Mantič *et al.*, 2020
Monocentrota lundstromi	MZH_HP.1540	ACJ8197	MZ631356	Roslin *et al.*, 2022
Platyura marginata	JSPM1	NULL	ON601503	Burdíková *et al.* 2024
Platyura pectoralis	JSK48	AEG7654	MT446919	Mantič *et al.*, 2020
Proceroplatus sp. 1	JSK25	AEG5475	MT446908	Mantič *et al.*, 2020
Proceroplatus sp. 2	JSK83	AEG8889	MT446938	Mantič *et al.*, 2020
Proceroplatus trinidadensis	NULL	ADY3950	MH998240	NULL
Proceroplatus biemarginatus **sp. n.**	NL-9-48	AEJ8294	PQ530757	This study
	NL-9-52	AEJ8294	PQ530758	
Proceroplatus dapanshana **sp. n.**	DPS-2-1–DPS-2-13	NULL	PQ530759–PQ530771	This study
	LWS-2-32	AEJ2594	PQ530772	
	LWS-2-55	AEJ2594	PQ530773	
	LWS-2-63	AEJ2594	PQ530774	
	LWS-2-76	AEJ2594	PQ530775	
	LWS-2-77	AEJ2594	PQ530776	
	LWS-2-92	AEJ2594	PQ530777	
	LWS-2-52	AEJ2594	PQ530778	
	LWS-3-5	AEJ2594	PQ530779	
	LWS-2-53	AEJ2594	PQ530780	
	LWS-2-31	AEJ2594	PQ530781	
	LWS-2-59	AEJ2594	PQ530782	
	LWS-2-61	AEJ2594	PQ530783	
	LWS-2-62	AEJ2594	PQ530784	
	LWS-2-64	AEJ2594	PQ530785	
	LWS-2-66	AEJ2594	PQ530786	
	LWS-2-68	AEJ2594	PQ530787	
	LWS-2-70–LWS-2-72	AEJ2594	PQ530788–PQ530790	
	LWS-2-80	AEJ2594	PQ530791	
	LWS-2-83	AEJ2594	PQ530792	
	LWS-2-93	AEJ2594	PQ530793	
	LWS-2-95	AEJ2594	PQ530794	
	LWS-2-56	AEJ2594	PQ530795	
	LWS-2-30	AEJ2594	PQ530796	
	LWS-2-91	AEJ2594	PQ530797	
	LWS-2-78	AEJ2594	PQ530798	
	LWS-2-79	AEJ2594	PQ530799	
	LWS-2-75	AEJ2594	PQ530800	

	LWS-2-81	AEJ2594	PQ530801
	LWS-2-84	AEJ2594	PQ530802
	LWS-2-60	AEJ2594	PQ530803
	LWS-3-1–LWS-3-4	AEJ2594	PQ530804–PQ530807
	LWS-2-94	AEJ2594	PQ530808
	LWS-2-90–LWS-2-86	AEJ2594	PQ530809–PQ530813
	LWS-2-82	AEJ2594	PQ530814
	LWS-2-74	AEJ2594	PQ530815
	LWS-2-73	AEJ2594	PQ530816
	LWS-2-69	AEJ2594	PQ530817
	LWS-2-67	AEJ2594	PQ530818
	LWS-2-65	AEJ2594	PQ530819
	LWS-2-58	AEJ2594	PQ530820
	LWS-2-57	AEJ2594	PQ530821
	LWS-2-51	AEJ2594	PQ530822
	LWS-2-85	AEJ2594	PQ530823
	LWS-2-43	AEJ2594	PQ530824
	TMS-2-50	AEJ2594	PQ530825
	LGS-10-40	AEJ2594	PQ530826
	LGS-10-41	AEJ2594	PQ530827
	DYS-10-64	AEJ2594	PQ530828
	DYS-10-67	AEJ2594	PQ530829
	FJS-10-29	AEJ2594	PQ530830
	FJS-10-31	AEJ2594	PQ530831
	GLG-8-4	AEJ2594	PQ530832
	DHS-8-89–DHS-8-86	AEJ2594	PQ530833–PQ530836
	DHS-8-79	AEJ2594	PQ530837
	DHS-8-75	AEJ2594	PQ530838
	DHS-8-74	AEJ2594	PQ530839
	DHS-8-62	AEJ2594	PQ530840
	HHG-7-90	AEJ2594	PQ530841
	HHG-7-91	AEJ2594	PQ530842
	WGS-9-5	AEJ2594	PQ530843
	WGS-9-6	AEJ2594	PQ530844
	WGS-9-9	AEJ2594	PQ530845
	WGS-9-20–WGS-9-23	AEJ2594	PQ530846–PQ530849
	WGS-9-16	AEJ2594	PQ530850
	WGS-9-18	AEJ2594	PQ530851
	WGS-9-19	AEJ2594	PQ530852
	GXHP-40	AEJ2594	PQ530853

References

Burdíková, N., Kaspřák, D., Kjærandsen, J., Tóthová, A. Š. & Ševčík, J. (2024) Molecular phylogeny of the fungus gnat subfamilies Sciophilinae and Leiinae (Mycetophilidae), with notes on Sciaroidea incertae sedis (Diptera: Bibionomorpha). *Zoological Journal of the Linnean Society*, 202 (1), zlad176.

Mantič, M., Sikora, T., Burdíková, N., Blagoderov, V., Kjærandsen, J., Kurina, O. & Ševčík, J. (2020) Hidden in plain sight: Comprehensive molecular phylogeny of Keroplatidae and Lygistorrhinidae (Diptera) reveals parallel evolution and leads to a revised family classification. *Insects*, 11 (6), 348.

Roslin, T., Somervuo, P., Pentinsaari, M., Hebert, P.D., Agda, J., Ahlroth, P., ... & Mutanen, M. (2022) A molecular-based identification resource for the arthropods of Finland. *Molecular Ecology Resources*, 22 (2), 803–822.

Ševčík, J., Hippa, H. & Burdíková, N. (2021) Just a fragment of undescribed diversity: Twenty new oriental and palearctic species of Sciaroidea (Diptera), including DNA sequence data and two new fossil genera. *Insects*, 13 (1), 19.
