# Supplementary material for: A Review of the Newly Recorded Genus Proceroplatus Edwards, 1925 (Diptera: Keroplatidae) in China with Two New Species, and Its Characterization and Phylogenetic Implication of Mitogenomes
Source: Insects. 2025 Aug 25;16(9):883. doi: 10.3390/insects16090883 (PMC12471120; doi:10.3390/insects16090883)
Supplement: Supplementary file 1 [file insects-16-00883-s001.zip › File S2.pdf]

File S2. GenBank accession numbers of sciaroid mitogenomes used in this study.

Number	Species	Family	GenBank No.	Reference
1	*Mayetiola destructor*	Cecidomyiidae	GQ387648	Beckenbach & Joy 2009
2	*Rhopalomyia pomum*	Cecidomyiidae	GQ387649	Beckenbach & Joy 2009
3	*Orseolia oryzae*	Cecidomyiidae	KM888183	Atray *et al.* 2015
4	*Allodia protenta*	Mycetophilidae	MN401738	Magnussen *et al.* 2022
5	*Tarnania fenestralis*	Mycetophilidae	MT410782	NULL
6	*Mycomya* sp.	Mycetophilidae	MW116802	Wang *et al.* 2021
7	*Acnemia nitidicollis*	Mycetophilidae	MT410840	NULL
8	*Azana* sp.	Mycetophilidae	MW116798	Wang *et al.* 2021
9	*Arachnocampa flava*	Keroplatidae	JN861748	Beckenbach 2012
10	*Proceroplatus dapanshanus* **sp. n.**	Keroplatidae	PV739439	This study
11	*Orfelia* sp.	Keroplatidae	MW394227	NULL
12	*Bradysia impatiens*	Sciaroidea	MZ202360	Wang *et al.* 2022
13	*Dolichosciara megumiae*	Sciaroidea	MN161588	Miao *et al.* 2020
14	*Pseudolycoriella* sp.	Sciaroidea	MN161587	Miao *et al.* 2020
15	*Sciara ruficauda*	Sciaroidea	MN161586	Miao *et al.* 2020
16	*Trichosia lengersdorfi*	Sciaroidea	MN161589	Miao *et al.* 2020

References

Atray, I., Bentur, J. S. & Nair, S. (2015) The Asian rice gall midge (*Orseolia oryzae*) mitogenome has evolved novel gene boundaries and tandem repeats that distinguish its biotypes. *PloS One*, 10 (7), e0134625.

Beckenbach, A. T. & Joy, J. B. (2009) Evolution of the mitochondrial genomes of gall midges (Diptera: Cecidomyiidae): rearrangement and severe truncation of tRNA genes. *Genome Biology and Evolution*, 1, 278–287.

Beckenbach, A. T. (2012) Mitochondrial genome sequences of *Nematocera* (lower Diptera): evidence of rearrangement following a complete genome duplication in a winter crane fly. G*enome Biology and Evolution*, 4 (2), 89–101.

Miao, X., Huang, J., Menzel, F., Wang, Q., Wei, Q., Lin, X. L. & Wu, H. (2020) Five mitochondrial genomes of black fungus gnats (Sciaridae) and their phylogenetic implications. *International Journal of Biological Macromolecules*, 150, 200–205.

Magnussen, T., Johnsen, A., Kjærandsen, J., Struck, T. H. & Søli, G. E. (2022) Molecular phylogeny of *Allodia* (Diptera: Mycetophilidae) constructed using genome skimming. *Systematic Entomology*, 47 (1), 267–281.

Wang, Q., Huang, J. & Wu, H. (2021) Mitogenomes provide insights into the phylogeny of Mycetophilidae (Diptera: Sciaroidea). *Gene*, 783, 145564.

Wang, Y., Liu, C., Wang, Q., Wu, H. & Huang, J. (2022) The complete mitochondrial genome of *Bradysia impatiens* (Diptera: Sciaridae). *Mitochondrial DNA Part B*, 7 (6), 1140–1142.
