# Supplementary material for: A Review of the Newly Recorded Genus Proceroplatus Edwards, 1925 (Diptera: Keroplatidae) in China with Two New Species, and Its Characterization and Phylogenetic Implication of Mitogenomes
Source: Insects. 2025 Aug 25;16(9):883. doi: 10.3390/insects16090883 (PMC12471120; doi:10.3390/insects16090883)
Supplement: Supplementary file 1 [file insects-16-00883-s001.zip › File S3.pdf]

File S3. World checklist of the genus *Proceroplatus*.

If not otherwise specified, the distribution of the species per country is provided according to Evenhuis (2006a).

***Proceroplatus aedon* (Vanschuytbroeck, 1965)**

Cerotelion aedon Vanschuytbroeck, 1965: 384.

Distribution. Cameroon, Central African Republic, Malawi, Tanzania, Zaire.

***Proceroplatus belluus* Matile, 1997**

Proceroplatus belluus Matile, 1997: 217.

Distribution: Panama.

***Proceroplatus bicornutus* (Matile, 1970)**

Orfelia (Proceroplatus) bicornutus Matile, 1970: 791.

Distribution: Cameroon.

***Proceroplatus biemarginatus* Wang *et* Huang, sp. n.**

Distribution: China: Guangdong (present paper).

***Proceroplatus borgmeieri* Shaw, 1940**

Platyura (*Proceroplatus*) *borgmeieri* Shaw, 1940: 803.

Distribution: Costa Rica.

***Proceroplatus catharinae* Edwards, 1932**

Platyura (*Proceroplatus*) *catharinae* Edwards, 1932: 139.

Distribution: Brazil.

***Proceroplatus dapanshanus* Wang *et* Huang, sp. n.**

Distribution: China: Guangxi, Guangdong, Guizhou, Zhejiang (present paper).

***Proceroplatus elegans* (Coquillett, 1895)**

Platyura elegans Coquillett, 1895: 307.

Distribution: Canada, USA.

***Proceroplatus graphicus* (Skuse, 1888)**

Platyura graphicus Skuse, 1888: 1179.

Distribution: Australia, Fiji (Evenhuis, 2006b).

***Proceroplatus guayanasi* Lane, 1950**

Platyura (*Proceroplatus*) *guayanasi* Lane, 1950: 59.

Distribution: Brazil.

***Proceroplatus hennigi*† Schumalfuss, 1979**

Proceroplatus hennigi† Schumalfuss, 1979: 4.

Distribution: Dominican.

***Proceroplatus iaunai* Lane, 1956**

Platyura (*Proceroplatus*) *iaunai* Lane, 1956: 126.

Distribution: Brazil.

***Proceroplatus juberthiei* Matile, 1982**

Proceroplatus juberthiei Matile, 1982: 215.

Distribution: Guadeloupe.

***Proceroplatus kerteszi* Lane, 1956**

Platyura elegans Kertesz, 1901: 404. Preoccupied by *P. elegans* Coquillett, 1895.
Platyura (*Proceroplatus*) *kerteszi* Lane, 1956: 125.

Distribution: Peru.

***Proceroplatus limpidapex* (Edwards, 1931)**

Platyura (*Proceroplatus*) *limpidapex* Edwards, 1931: 487.

Distribution: Indonesia.

***Proceroplatus mikado* (Okada, 1938)**

Zelmira mikado Okada, 1938: 27.

Distribution: Japan.

***Proceroplatus minutus* Matile, 1974**

Proceroplatus minutus Matile, 1974: 925.

Distribution: Central African.

***Proceroplatus moala* Evenhuis, 2006**

Proceroplatus moala Evenhuis, 2006b: 7.

Distribution: Fiji.

***Proceroplatus paramariboensis* Edwards, 1934**

Platyura (*Proceroplatus*) *paramariboensis* Edwards, 1934: 356.

Distribution: Surinam, Costa Rica, Trinidad.

***Proceroplatus paucimaculatus* Matile, 1998**

Proceroplatus paucimaculatus Matile, 1998: 120.

Distribution: Namibia.

***Proceroplatus pectinatus* Evenhuis, 2006**

Proceroplatus pectinatus Evenhuis, 2006b: 4.

Distribution: Fiji.

***Proceroplatus pictipennis* Williston, 1896**

Platyura pictipennis Williston, 1896: 257.

Proceroplatus pictipennis Edwards, 1925: 523.

Platyura (*Proceroplatus*) Edwards, 1929: 171.

Distribution: Mexico, British Virgin Island, Trinidad, Hispaniola (Penney *et al.* 2013).

***Proceroplatus pictus* (Speiser, 1908)**

Ceroplatus pictus Speiser, 1908: 128.

Distribution: Tanzania.

***Proceroplatus poecilopterus* Edwards, 1927**

Proceroplatus poecilopterus Edwards, 1927: 117.

Distribution: Sri Lanka.

***Proceroplatus preziosii*† Evenhuis & Penney, 2013**

Proceroplatus preziosii† Evenhuis & Penney, 2013: 595.

Distribution: Dominicana (Penney *et. al*, 2013).

***Proceroplatus priapus* Matile, 1988**

Proceroplatus priapus Matile, 1988: 117.

Distribution: New Caledonia, Fiji (Evenhuis, 2006b).

***Proceroplatus pulchripennis* (Senior-White, 1922)**

Ceroplatus pulchripennis Senior-White, 1922: 195.

Distribution: Sri Lanka.

***Proceroplatus puncticoxalis* Edwards, 1940**

Platyura (Proceroplatus) puncticoxallis Edwards, 1940: 451.

Distribution: Brazil, Argentina.

***Proceroplatus rabelloi* Lane, 1956**

Platyura (*Proceroplatus*) *rabelloi* Lane, 1956: 127.

Distribution: Brazil, Peru.

***Proceroplatus scalprifera* Matile, 1988**

Proceroplatus scalprifera Matile, 1988: 118.

Distribution: New Caledonia, Fiji (Evenhuis, 2006b).

***Proceroplatus seguyi* (Matile, 1972)**

Orfelia (Proceroplatus) seguyi Matile, 1972: 115;

Orfelia (Proceroplatus) mirabilis Matile, 1972: 115;

Distribution: Reunion.

***Proceroplatus stonei* (Lane, 1950)**

Platyura (*Proceroplatus*) *stonei* Lane, 1950: 60.

Proceroplatus stonei (Lane): Papavero, 1978: 11.

Distribution: USA, Dominica, Guadeloupe, Jamaica, Puerto Rico.

***Proceroplatus suffusinervis* (Brunetti, 1912)**

Platyura suffusinervis Brunetti, 1912: 60.

Distribution: Indonesia (Sabah).

***Proceroplatus terenoi* Lane, 1950**

Platyura (Proceroplatus) terenoi Lane, 1950: 61.

Distribution: Brazil, Argentina.

***Proceroplatus trinidadensis* (Lane, 1960)**

Platyura (*Proceroplatus*) *trinidadensis* Lane, 1960: 379.

Proceroplatus trinidadensis (Lane): Papavero, 1978: 11.

Distribution: Trinidad, Ecuador, Panama, Netherlands (Chandler & Pijnakker 2009), Westland (Chandler & Pijnakker 2009), France (Gloaguen, 2022).

***Proceroplatus variventris* Edwards, 1931**

Platyura (*Proceroplatus*) *variventris* Edwards,1931: 76.

Distribution: Bolivia.

***Proceroplatus venustipennis* Shaw, 1940**

Platyura (Proceroplatus) venustipennis Shaw, 1940: 804.

Distribution: Costa Rica.

***Proceroplatus vilasboasi* Lane, 1961**

Platyura (*Proceroplatus*) vilasboasi Lane, 1961: 5.

Distribution: Brazil.

***Proceroplatus vittatus* Fisher, 1941**

Platyura (Proceroplatus) vittata Fisher, 1941: 8.

Distribution: Costa Rica.

***Proceroplatus whitfieldii* Matile, 1974**

Proceroplatus whitfieldii Matile, 1974: 926.

Distribution: Central African.

***Proceroplatus zeijsti* Matile, 1988**

Proceroplatus zeijsti Matile, 1988: 68.

Distribution: Ivory Coast.

References

Brunetti, E. (1912) *Diptera Nematocera (excluding Chironomidae and Culicidae). Vol. 1*. Taylor & Francis, London, 574 pp.

Chandler, P.J. & Pijnakker, J. (2009) Tropical fungus gnats established in nurseries in The Netherlands (Diptera: Keroplatidae) and Mycetophilidae. *British Journal of Entomology and Natural History*, 22 (2), 81.

Coquillett, D.W. (1895) New North American Mycetophilidae. *Canadian Entomologist*, 27, 199–201.

Edwards, F.W. (1927) Some Nematocerous Diptera from Ceylon. *Spolia Zeylaniea*, 211.

Edwards. F.W. (1931) Diptera Nematocera from the lowlands of North Borneo. *Journal of the Federated Malay States Museums*, 486–504.

Edwards. F.W. (1932) New Brazilian Mycetophilidae (Diptera). *Revista de Entomologia*, 2, 138–149.

Edwards, F.W. (1934) New Neotropical Mycetophilidae (III) (Diptera). *Revista de Entomologia*, 4, 354–372.

Edwards, F.W. (1940) New Neotropical Mycetophilidae (IV) (Diptera). *Revista de Entomologia*, 11, 440–465, 2 pls.

Evenhuis, N.L. (2006a) Catalog of the Keroplatidae of the world (Insecta: Diptera). *Bishop Museum Bulletin in Entomology*, 13, 1–178.

Evenhuis N.L. (2006b) Two new species of *Proceroplatus* Edwards (Diptera: Keroplatidae) from Fiji. *Fiji Arthropods IV*, 86, 3–9.

Fisher, E.G. (1941) Notes on Costa Rican Mycetophilidae (Diptera). *Entomological News*, 52, 8.

Gloaguen, P.Y. (2022) Premier signalement du genre *Proceroplatus* Edwards, 1925 en France métropolitaine (Diptera Keroplatidae). *L'Entomologiste*, 78 (3), 161–165.

Lane, J. (1950) Neotropical Ceroplatinae (Diptera, Mycetophilidae). *Dusenia*, 1 (1), 32–69.

Lane, J. (1956) Further notes on Neotropical Keroplatinae (Diptera, Mycetophilidae). *Revista Brasileira de Biologia*, 16, 121–128.

Lane, J. (1960) Mycetophilidae from Trinidad, B. W. I. (Diptera, Nematocera). *Studia Entomologica*, 3, 375–384.

Lane, J. (1961) Further new Neotropical Mycetophilidae (Diptera, Nematocera). *RevistaBrasileira de Entomologia*, 10, 1–15.

Matile, L. (1970) Diptères Mycetophilidae du Cameroun et de République centrafricaine. *Bulletin de l'Institut Fondamental d'Afrique Noire*, 773–816.

Matile, L. (1972) Keroplatinae des iles de l'Océan Indien occidental (Diptera, Mycetophilidae). *Cahiers O.R.S.T.O.M.* (Série Biologie), 16, 105–123.

Matile L. (1974) South African Animal Life. IX. Diptera Mycetophilidae Keroplatinae. South African Animal Life. *Stockholn1*, 15, 511–532.

Matile, L. (1982) Systématique, phylogénie et biogéographie des Diptères Keroplatidae des Petites Antilles et de Trinidad. *Bulletin du Museum National d'Histoire Naturelle (A)*, 4 (1-2), 189–235.

Matile, L. (1988) Diptères Mycetophiloidea de Nouvelle-Calédonie. 2. Keroplatidae. *Mémoires du Muséum national d'Histoire naturelle*, 142, 89–136.

Matile, L. (1997) Keroplatidae de la savane arbustive de Namibie [Diptera, Sciaroidea]. *Revue française d'entomologie (1979)*, 19 (3-4), 117–121.

Okada, I. (1938) Beitrag zur kenntnis der Ceroplatinen-Fauna Japans (Diptera, Fungivoridae). *Insecta Matsumurana*, 13 (1), 17–32.

Penney, D., Evenhuis, N.L., & Green, D.I. (2013) A new species of *Proceroplatus* Edwards (Diptera: Keroplatidae) in Miocene amber from the Dominican Republic. *Zootaxa*, 3686, 593–599.

Schmalfuss H. (1979) *Proceroplatus hennigi* n. sp., die erste Pilzmücke aus dem Dominikanischen Bernstein (Stuttgarter Bernsteinsammlung: Diptera, Mycetophiloidea, Keroplatidae). *Stuttgarter Beiträge zur Naturkunde Serie B* (Geologie und Paläontologie), 49, 1–9.

Senior-white, R.A. (1921) New ceylon diptera (Part 11.). *Spolia Zeylanica*, XII (45), 195–206.

Shaw, F.R. (1940) Some new Mycetophilidae from Costa Rica (Diptera). Part 1. *Rev. de Entomologia*, vol. 11, 803–808.

Skuse, F.A.A. (1888) Diptera of Australia. Part III.—The Mycetophilidae. *Proceedings of the Linnean Society of New South Wales*, (2) 3: 1123–1222.

Speiser, P. (1908) Dipteren aus Deutschlands afrikanischen Kolonien. *Berliner Entomologische Zeitschrift*, 52 (3), 127.

Vanschuytbroeck, P. (1965) Mission zoologique de I'I.R.S.A.C. en Afrique orientale. (P. Basilewsky et N. Leleup, 1957). XCVIII. Diptera Mycetophilidae et Anisopodidae. *Annales du Muséum Royal d'Afrique Centrale (Séries 8vo) (Zoologie)*, 138, 381–393.

Williston, S.W. (1896) On the Diptera of St. Vincent (West Indies). *Transactions of the Entomological Society of London*, 1896, 253–446.
