# Supplementary material for: A Review of the Newly Recorded Genus Proceroplatus Edwards, 1925 (Diptera: Keroplatidae) in China with Two New Species, and Its Characterization and Phylogenetic Implication of Mitogenomes
Source: Insects. 2025 Aug 25;16(9):883. doi: 10.3390/insects16090883 (PMC12471120; doi:10.3390/insects16090883)

File S4. Prediction of tRNA secondary structures in the mitogenome of *Proceroplatus dapanshanus* **sp. n.**

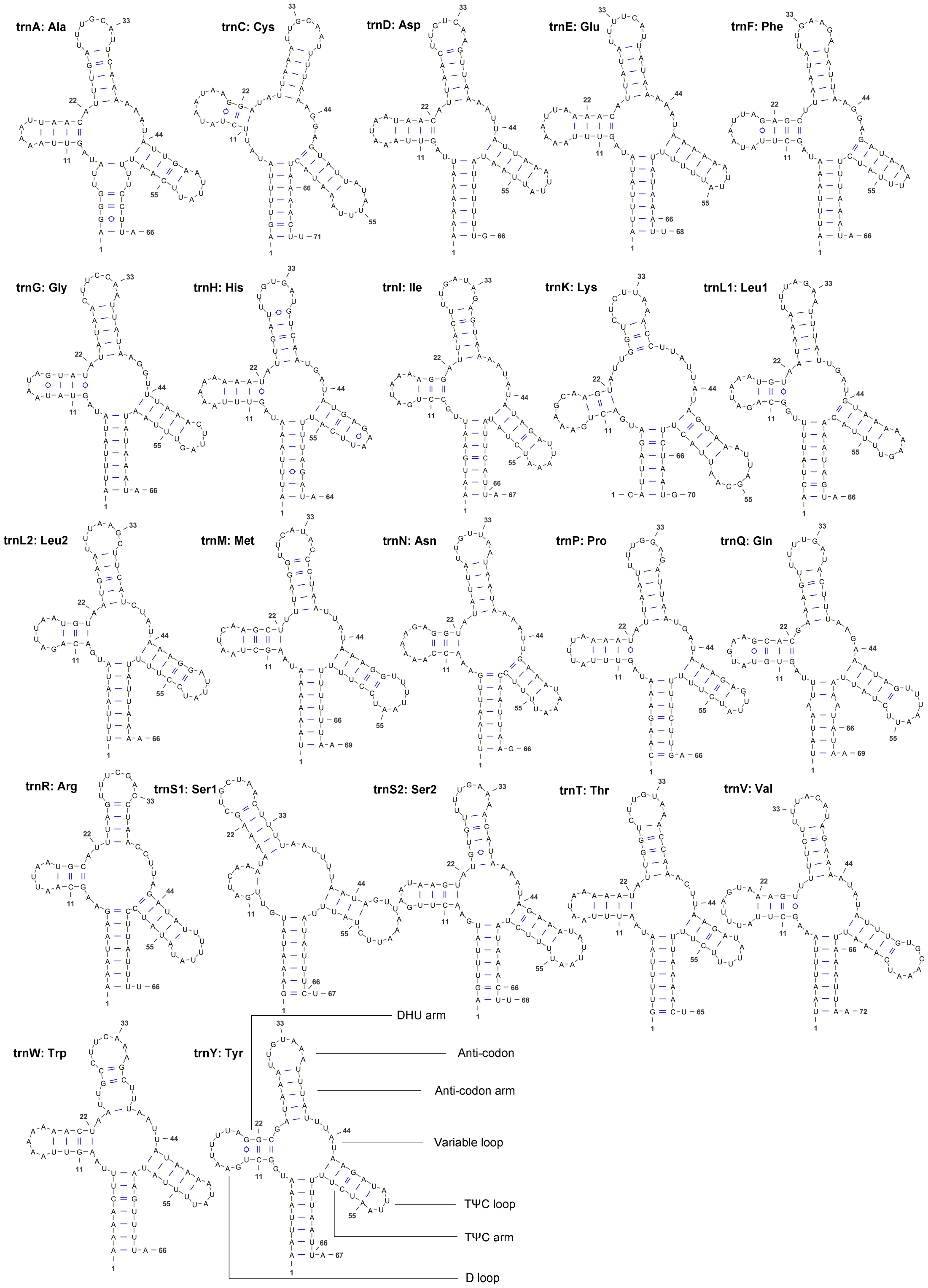

Supplement: Supplementary file 1 [file insects-16-00883-s001.zip › File S4.pdf]
