# Supplementary material for: A Review of the Newly Recorded Genus Proceroplatus Edwards, 1925 (Diptera: Keroplatidae) in China with Two New Species, and Its Characterization and Phylogenetic Implication of Mitogenomes
Source: Insects. 2025 Aug 25;16(9):883. doi: 10.3390/insects16090883 (PMC12471120; doi:10.3390/insects16090883)
Supplement: Supplementary file 1 [file insects-16-00883-s001.zip › File S5.pdf]

File S5. ENc-plot, PR2-plot, and neutrality-plot results of three keroplatid mitogenomes.

Species	PCG	ENc	G3/(G3+C3)	A3/(A3+T3)	GC12	GC3
Arachnocampa flava	atp6	31.80	0.048	0.449	0.301	0.092
	atp8	/	0.000	0.481	0.173	0.055
	cox1	33.43	0.286	0.507	0.380	0.083
	cox2	31.49	0.111	0.385	0.315	0.079
	cox3	31.80	0.154	0.441	0.337	0.061
	cytb	29.50	0.353	0.421	0.325	0.061
	nad1	31.25	0.786	0.448	0.279	0.063
	nad2	26.67	0.214	0.425	0.196	0.059
	nad3	33.17	0.214	0.448	0.263	0.110
	nad4	32.32	0.813	0.495	0.261	0.077
	nad4l	27.98	1.000	0.511	0.177	0.081
	nad5	31.70	0.706	0.498	0.249	0.067
	nad6	26.44	0.333	0.468	0.184	0.017
Orfelia sp.	atp6	38.43	0.158	0.500	0.328	0.086
	atp8	/	0.333	0.423	0.104	0.075
	cox1	32.02	0.292	0.492	0.386	0.099
	cox2	37.38	0.217	0.480	0.316	0.105
	cox3	38.55	0.097	0.537	0.363	0.147
	cytb	33.38	0.200	0.505	0.333	0.116
	nad1	31.26	0.739	0.419	0.309	0.113
	nad2	32.49	0.257	0.484	0.236	0.123
	nad3	30.44	0.167	0.538	0.294	0.097
	nad4	35.09	0.636	0.422	0.266	0.102
	nad4l	36.08	0.923	0.420	0.239	0.120
	nad5	34.47	0.739	0.475	0.286	0.105
	nad6	36.90	0.111	0.536	0.207	0.088
Proceroplatus dapanshanus **sp. n.**	atp6	36.30	0.300	0.515	0.327	0.089
	atp8	/	0.000	0.453	0.137	0.036
	cox1	33.62	0.319	0.448	0.382	0.092
	cox2	32.99	0.100	0.422	0.323	0.089
	cox3	35.65	0.161	0.487	0.368	0.118
	cytb	35.76	0.265	0.494	0.353	0.129
	nad1	32.09	0.800	0.404	0.297	0.110
	nad2	32.53	0.333	0.429	0.214	0.052
	nad3	31.77	0.231	0.486	0.284	0.110
	nad4	34.37	0.614	0.459	0.266	0.099
	nad4l	25.21	0.800	0.447	0.227	0.050
	nad5	35.12	0.671	0.429	0.272	0.127
	nad6	29.73	0.286	0.497	0.199	0.081
